# Emotional Distress Symptom Networks in Patients with Gynecological Malignancies: A Cross-Sectional Study

**DOI:** 10.3390/healthcare14091136

**Published:** 2026-04-23

**Authors:** Haowen Huang, Ting Liu, La Pan, Shuo Man, Ling Xia, Yuan Wang

**Affiliations:** 1 Department of Gynecologic Oncology, Affiliated Hospital of Jiangnan University, Wuxi 214122, China; 6232807009@stu.jiangnan.edu.cn (H.H.); 6242807018@stu.jiangnan.edu.cn (L.P.); 2 Wuxi School of Medicine, Jiangnan University, Wuxi 214122, China; 6252807007@stu.jiangnan.edu.cn; 3 The Affiliated Hospital of Xuzhou Medical University, Xuzhou 221002, China; 6222807037@stu.jiangnan.edu.cn

**Keywords:** emotional distress, gynecological malignancies, symptom network analysis, fatigue, quality of life, psychosocial factors

## Abstract

**Background:** Emotional distress (ED) is common among patients with gynecological malignancies and is associated with reduced quality of life and suboptimal health outcomes. Total-score approaches may overlook the complex interrelationships among individual emotional symptoms. Objective: This study provides a theory-informed contextual application and empirical boundary test of symptom network analysis, organized by the Stress Process Model (SPM), to examine not only how ED symptoms cluster and connect with psychosocial correlates and quality-of-life domains, but also whether psychosocial stratification is reflected in altered symptom topology or primarily in differences in distress burden. **Methods:** A cross-sectional study was conducted among 415 patients with gynecological malignancies recruited from a tertiary hospital in China. ED was assessed using the Brief Profile of Mood States-Short Form (BPOMS-SF30). An exploratory three-track screening strategy was used to derive a focused 16-node set of frequent negative mood symptoms. Gaussian graphical models with EBICglasso regularization were estimated for the symptom network and for extended networks including demographic/clinical variables, SPM-related psychosocial variables, and quality-of-life indicators. **Results:** The ED network showed dense positive connectivity, with strong within-domain clustering and several cross-domain associations. Exhaustion, restlessness, and irritability were relatively more relationally prominent in the primary network, although centrality stability was low to moderate across models. Fatigue-related symptoms were closely connected with anxiety, depressive symptoms, and impaired quality of life. Among psychosocial variables, self-perceived burden showed the strongest conditional association with fatigue. Adjusting for demographic and clinical variables did not materially alter the core symptom network, and no significant subgroup differences in global strength or overall structure were observed across psychosocial strata. **Conclusions:** In this sample, psychosocial risk stratification appeared to relate more to the overall severity and burden of distress than to major reorganization of symptom topology. The study therefore contributes primarily as a theory-informed contextual application of network methods and as an empirical boundary test showing that several psychosocial strata did not exhibit major topological differences. Because the retained nodes were selected for prevalence, association strength, and selection stability, the observed prominence of fatigue- and activation-related symptoms should be interpreted as conditional on this focused symptom subset. Overall, the findings are correlational, exploratory, and hypothesis-generating.

## 1. Introduction

Gynecological malignancies are major threats to women’s health worldwide [1,2]. Emotional distress (ED) in oncology refers to persistent unpleasant emotional experiences related to cancer and its treatment, including anxiety, depression, fear, helplessness, and emotional dysregulation [3]. Across cancer populations, approximately 30–40% of patients experience moderate or severe distress [4,5], and rates in gynecologic oncology may be even higher because the disease can affect fertility, body image, sexuality, and gender-role expectations [6,7,8,9,10,11].

ED is associated with poorer quality of life, treatment burden, and adverse symptom experiences [12,13]. However, ED symptoms seldom occur in isolation. Anxiety, depression, fatigue, anger, and confusion often co-occur and reinforce one another, whereas total-score approaches may obscure these symptom-to-symptom dependencies [14,15,16].

Symptom network analysis conceptualizes symptoms as mutually related components within a system rather than as interchangeable indicators of a single latent construct [15,16,17,18]. This perspective can identify relatively prominent symptoms and cross-domain couplings that may be useful for descriptive clinical monitoring. Despite growing oncology applications, evidence in gynecologic malignancies remains limited, and studies rarely examine emotional distress symptoms together with psychosocial stressors and quality-of-life domains within the same network framework. In this study, we use network methods within an SPM-informed framework to examine how emotional distress symptoms are organized in gynecologic oncology and how they relate to stress-related psychosocial correlates. The Stress Process Model (SPM) provides a useful contextual framework for organizing these correlates [19,20]. In patients with gynecological malignancies, disease burden, treatment, and functional limitations may act as primary stressors, while illness perception, financial strain, role disruption, and self-perceived burden may operate as secondary stressors or stress-related correlates [21,22]. At the same time, SPM and network theory rest on different assumptions: SPM is often articulated as a directional and partially hierarchical process model, whereas Gaussian graphical models estimate undirected conditional dependence among contemporaneously measured variables. We therefore use the SPM to organize variable selection and interpretation at the level of stress exposure, psychosocial appraisal, and functioning. In the extended models, psychosocial variables are conceptualized as external correlates positioned alongside, rather than inside, the core symptom system for purposes of conditional association mapping. More specifically, the subgroup comparisons were intended to examine whether psychosocial stratification—central to the SPM—was accompanied by differences in how symptoms interrelated, or whether symptom organization remained broadly stable while overall distress burden varied.

Accordingly, this study should be understood as a theory-informed contextual application of network analysis in a gynecologic oncology sample rather than a formal extension of the SPM. We aimed to (1) characterize the conditional association structure of ED symptoms; (2) examine how demographic/clinical variables, SPM-related psychosocial variables, and quality-of-life domains are conditionally linked with ED symptoms; and (3) explore whether psychosocial risk strata differ in overall network topology, thereby bearing on the distinction between structural differentiation and severity variation.

## 2. Methods

### 2.1. Participants

This study adopted a cross-sectional design. Using a convenience sampling method, a total of 415 patients with gynecological malignancies were consecutively recruited from the gynecological wards of the Affiliated Hospital of Jiangnan University between 1 October 2024 and 31 May 2025. All participants met the inclusion criteria and voluntarily participated in the study. The research protocol was approved by the Ethics Committee of the Affiliated Hospital of Jiangnan University (Approval: No. LS2024284). All procedures were conducted in accordance with the Declaration of Helsinki.

The inclusion criteria were as follows: (1) histologically confirmed gynecological malignancy (including cervical, ovarian, endometrial, vulvar, or vaginal cancer); (2) age ≥ 18 years; (3) normal cognitive function with the ability to read and communicate in Chinese; (4) provision of written informed consent; and (5) having completed at least one major cancer treatment (surgery, chemotherapy, or radiotherapy) and currently being in the treatment or follow-up phase.

The exclusion criteria included the following: (1) a history of severe psychiatric disorders identified through medical records or self-report; (2) major comorbid conditions that could substantially interfere with emotional assessment; and (3) cognitive or language impairments that prevented completion of the questionnaires.

### 2.2. Sample Size

Based on the planned 16-node symptom network, requiring estimation of 16 threshold parameters and 96 pairwise association parameters [16 × (16 − 1)/2], yielding a total of 120 parameters. Following commonly used guidance for exploratory psychological networks, we aimed for approximately 3–5 participants per parameter, which yielded an estimated minimum sample size of 341–569 after allowing for 20% attrition [23].

### 2.3. Measurements

#### 2.3.1. General Information Questionnaire

A self-designed questionnaire was used to collect demographic and clinical information. Demographic variables included age, sex, ethnicity, marital status, body mass index (BMI), family economic status, educational level, and employment status. Clinical variables included age at menarche, reproductive history, family history of cancer, surgical history, cancer diagnosis, duration since diagnosis, treatment modality, tumor stage, and comorbid medical conditions.

#### 2.3.2. Brief Profile of Mood States—Short Form (BPOMS-SF30)

The BPOMS-SF30 is a widely used instrument for assessing mood and emotional states [24]. The scale consists of 30 adjectives assessing psychological states over the past week across six dimensions: tension/anxiety, fatigue, vigor, confusion, depression, and anger. Each item is rated on a 5-point Likert scale ranging from 0 (“not at all”) to 4 (“extremely”), with higher total scores indicating greater emotional distress. In this study, the Cronbach’s α coefficient for the BPOMS-SF30 was 0.742.

#### 2.3.3. Brief Illness Perception Questionnaire (IPQR)

The Chinese version of the IPQR has demonstrated acceptable reliability and validity in oncology populations [25]. The questionnaire contains nine items assessing cognitive representation (items 1–5), emotional representation (items 6 and 8), and illness comprehensibility (item 7). Items are scored on an 11-point scale (0–10), with items 3, 4, and 7 reverse-coded. The total score ranges from 0 to 80, with higher scores indicating more negative illness perceptions. Item 9 is an open-ended question and is not scored. In this study, the Cronbach’s α coefficient was 0.721.

#### 2.3.4. Neuroticism Subscale of the Big Five Inventory (BFI-N)

Neuroticism was assessed using the neuroticism subscale of the Big Five Inventory [26]. The subscale consists of eight items rated on a 5-point Likert scale, with items 2, 5, and 7 reverse-coded. Higher scores indicate higher levels of neuroticism. The validated Chinese version was used in this study, yielding a Cronbach’s α coefficient of 0.920.

#### 2.3.5. Positive Psychological Capital Questionnaire (PPQ)

Positive psychological capital was measured using the Positive Psychological Capital Questionnaire revised by Zhang Kuo et al. [27]. The questionnaire consists of 26 items across four dimensions: self-efficacy (7 items), resilience (7 items), hope (6 items), and optimism (6 items). Items are rated on a 5-point Likert scale, with items 8, 10, 12, 14, and 25 reverse-coded. Higher scores indicate higher levels of positive psychological capital. The Cronbach’s α coefficient in this study was 0.725.

#### 2.3.6. Comprehensive Score for Financial Toxicity Based on Patient-Reported Outcome Measure (COST-PROM)

Financial toxicity was assessed using the Chinese version of the COST-PROM [21]. The scale consists of 11 items covering financial expenditure, financial resources, and social response. Each item is scored from 0 to 4, with items 2, 3, 4, 5, 8, 9, and 10 reverse-coded. Total scores range from 0 to 44, with lower scores indicating more severe financial toxicity. In this study, the Cronbach’s α coefficient was 0.743.

#### 2.3.7. Self-Perceived Burden Scale (SPBS)

The SPBS was translated and validated for use in China and has been widely applied among patients with chronic diseases [28]. The scale includes 10 items measuring physical burden (items 1 and 10), emotional burden (items 2, 4, 5, 6, 7, and 9), and financial burden (items 3 and 8). Items are rated on a 5-point Likert scale ranging from 1 (“never”) to 5 (“always”), with higher scores indicating greater perceived burden. Total scores classify burden as none (<20), mild (20–29), moderate (30–39), or severe (≥40). The Cronbach’s α coefficient in this study was 0.744.

#### 2.3.8. European Organization for Research and Treatment of Cancer Quality of Life Questionnaire (EORTC QLQ-C30)

Quality of life was assessed using the Chinese version of the EORTC QLQ-C30 [29]. The questionnaire comprises 30 items covering five functional domains, three symptom domains, one global health status/quality-of-life domain, and six single items. Items 29 and 30 are rated on a 7-point scale, while the remaining items use a 4-point scale. In this study, functional domain scores and the global health status score were calculated and linearly transformed to standardized scores ranging from 0 to 100, with higher scores indicating better quality of life. The Cronbach’s α coefficient was 0.769.

#### 2.3.9. Statistical Analysis

Descriptive statistics were performed using IBM SPSS Statistics 28.0, and network analyses were conducted in R version 4.4.2.

Descriptive statistics were used to summarize demographic and clinical characteristics and emotional distress levels. Normally distributed continuous variables are presented as mean ± standard deviation (SD), non-normally distributed continuous variables as median (Q1, Q3), and categorical variables as frequencies and percentages.

Missing values were handled using multiple imputation by chained equations with predictive mean matching implemented in the mice package in R [30]. Imputation was used as a preprocessing step to reduce information loss before the exploratory network analyses.

Before network estimation, BPOMS-SF30 items were screened to identify a focused set of clinically frequent negative mood symptoms. The total sample was randomly divided into training and validation sets using a fixed random seed, and screening was performed in the training set only. Because this procedure was exploratory and data-informed rather than confirmatory, the resulting reduced network should be interpreted as a focused symptom subset rather than a full representation of the BPOMS-SF30 construct space. The screening strategy was not preregistered and should therefore be regarded as an exploratory analytic step. Three complementary approaches were combined: (1) Spearman correlation analysis with the core distress indicator, retaining items with |ρ| ≥ 0.30 and a positive response rate ≥ 0.50; (2) 10-fold cross-validated penalized regression with continuous distress as the outcome; and (3) repeated cross-validated L1-penalized logistic regression using a dichotomized distress outcome, with items retained if selected in ≥60% of repetitions [31,32,33]. Items were combined across approaches using predefined weights (1/2/2), and those meeting the overall criteria were retained. In addition to these statistical filters, the final node set was reviewed for clinical interpretability and for its coverage of frequently endorsed negative mood symptoms; excluded items should not be interpreted as clinically irrelevant.

To minimize redundancy, item pairs with Spearman correlations > 0.80 were examined, and the more informative items were retained as final symptom nodes. The selected node set was then frozen and evaluated in the validation set and the full sample, resulting in a final 16-node network. Primary networks were estimated using Gaussian graphical models (GGMs) with EBICglasso regularization [23,34,35]. Edges represent regularized partial correlations after conditioning on all other variables. Networks were visualized using the qgraph (v1.9.8) qgraph package [35].

Centrality analyses focused on node strength and expected influence (EI), because closeness and betweenness are often unstable in psychological networks [23,34]. Network accuracy and stability were evaluated using nonparametric bootstrap procedures with 1000 resamples, including 95% confidence intervals for edge weights and correlation stability (CS) coefficients for centrality metrics. Given that several CS coefficients were below ideal thresholds, centrality rankings were interpreted descriptively and cautiously. Apparent differences among the top-ranked nodes should therefore not be interpreted as statistically established unless supported by dedicated bootstrapped centrality difference testing.

Extended network analyses were conducted by including psychosocial variables as external nodes to examine their conditional associations with emotional symptoms. In addition, residual networks were estimated by regressing each symptom on demographic and clinical covariates, extracting residuals, applying nonparanormal transformations, and re-estimating the networks using EBICglasso (γ = 0.50). Centrality was compared across models.

Group differences in network topology were examined using the Network Comparison Test (NCT). Participants were dichotomized at the median of each SPM-related variable, and differences in global strength and network structure were assessed using 1000 permutation tests. Because median splits may reduce statistical power, subgroup results were interpreted as exploratory.

## 3. Results

### 3.1. Participant Characteristics

A total of 415 patients with gynecologic malignancies were included. The mean age was 59.4 years, indicating that the sample was predominantly middle-aged to older adults. Additional demographic and clinical characteristics are presented in Table 1.

### 3.2. Node Screening and Validation

**Figure 1:**
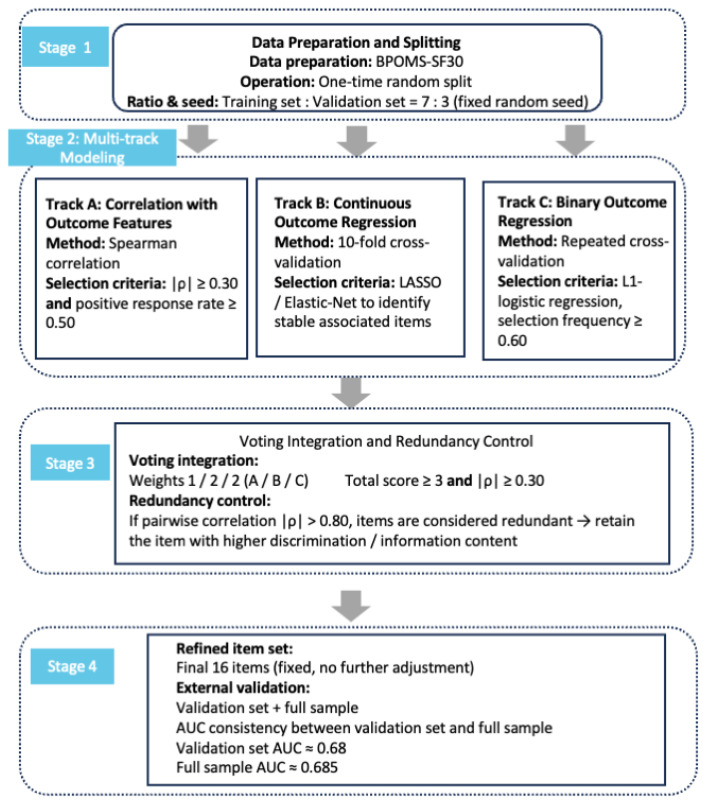
Three-Track Parallel Screening Strategy for Symptom Selection.

Node screening was completed in the training set using a three-track parallel strategy. Based on symptom prevalence, 23 symptoms with an occurrence rate > 50% were entered into the screening process [23]. After redundancy control, 16 emotional symptom nodes were retained and subsequently frozen. These retained nodes represented frequently endorsed negative mood symptoms and were kept unchanged in all subsequent analyses (Figure 1). The final frozen nodes covered five negative mood dimensions: Tension–Anxiety (TA2–TA4), Depression–Dejection (DD1–DD4), Anger–Hostility (AH2), Fatigue–Inertia (FI1–FI5), and Confusion–Bewilderment (CB1–CB3). The frozen node set demonstrated acceptable discrimination in the validation set (AUC = 0.680) and the full sample (AUC = 0.685), supporting the robustness of the screening results while still representing a reduced subset rather than the full BPOMS-SF30. As a sensitivity analysis, we reconstructed the network using the 23 symptom items retained after prevalence filtering alone and compared it with the screened 16-node network. The two networks showed broadly consistent overall topology and core structural patterns, indicating that the main findings were not driven entirely by the final screening step (Appendix A).

To assess stability and reproducibility across samples, descriptive consistency testing was performed after node freezing. Specifically, the mean scores and positive response proportions (defined as node score ≥ a prespecified threshold) were calculated for each node in the training and validation sets and compared. Overall, node means and positive response proportions were broadly comparable between the two sets, with no clear systematic shifts, suggesting good distributional stability (Table 2).

### 3.3. Emotional Distress Symptom Network

**Figure 2:**
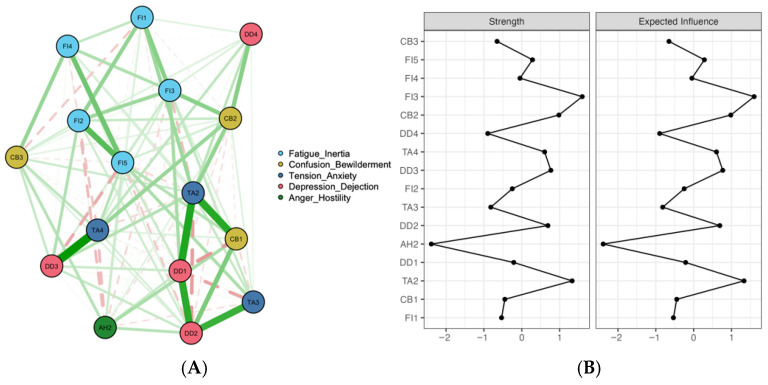
Network analysis visualization for emotional distress (**A**) and centrality metrics (**B**). Note: Green edges indicate positive partial correlations and red edges indicate negative partial correlations; thicker lines indicate stronger absolute edge weights. Centrality plots present z-standardized strength and expected influence (EI).

The emotional distress symptom network estimated by EBICglasso showed a dense structure dominated by positive edges, indicating predominantly reinforcing conditional associations among symptoms (Figure 2A). The network included 16 nodes and 66 nonzero edges (out of 120 possible edges). Clear within-dimension clustering was observed, with stronger connections within the same mood dimension and fewer cross-dimension links. The strongest edge was observed between TA4 (Anxious) and FI4 (Cannot get going) (ω = 0.383). Additional relatively strong edges included CB2 (Drowsy) with TA2 (Restless) (ω = 0.265) and DD4 (Blue) with TA2 (Restless) (ω = 0.244), suggesting meaningful cross-domain couplings. Centrality analysis suggested that FI3 (Exhausted) (rs = 1.60; rEI = 1.60), TA2 (Restless) (rs = 1.33; rEI = 1.33), and AH2 (Irritable) (rs = 2.39; rEI = 2.39) were relatively more central than other nodes (Figure 2B). Because the case-dropping bootstrap produced a CS coefficient of 0.27 (cor = 0.7), these centrality rankings should be interpreted cautiously and primarily as descriptive.

### 3.4. Expanded Network with Demographic and Clinical Variables

**Figure 3:**
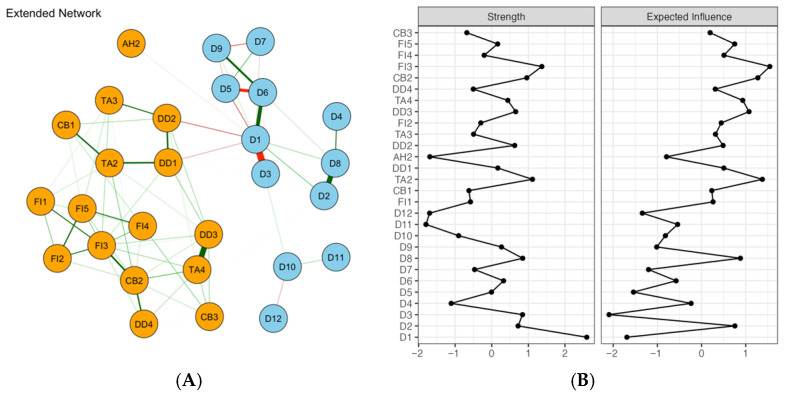
Network analysis visualization for emotional distress with demographic and clinical variables (**A**) and centrality metrics (**B**). Note: Green edges indicate positive partial correlations and red edges indicate negative partial correlations; thicker lines indicate stronger absolute edge weights. Centrality plots present z-standardized strength and expected influence (EI). D1 = age group; D2 = marital status; D3 = type of menopause; D4 = parity; D5 = educational level; D6 = employment status; D7 = monthly income level; D8 = primary caregiver; D9 = type of medical insurance; D10 = surgical approach; D11 = tumor stage; D12 = treatment modality.

As shown in Figure 3A, an expanded association network was constructed incorporating emotional symptoms and demographic/clinical variables. The model included 28 nodes and 94 nonzero edges (out of 378 possible edges). Age group (D1), monthly income (D7), and tumor stage (D11) exhibited comparatively more connections with emotional symptoms, whereas marital status (D2), parity (D4), and insurance type (D9) showed fewer links. The three strongest edges by absolute weight were education level (D5)-type of surgery (D10) (ω = −0.537), insurance type (D9)-TA2 (Restless) (ω = 0.309), and tumor stage (D11)-TA2 (Restless) (ω = 0.300). After removing covariate effects (residual network; Figure 3B), the overall symptom network structure changed minimally. Centrality results in this expanded network indicated that TA2 (Restless), FI3 (Exhausted), and age group (D1) had relatively higher centrality; however, given the CS coefficient of 0.20 (cor = 0.7), these rankings should be interpreted only descriptively.

### 3.5. Expanded Network with Stress Process Model–Related Variables

**Figure 4:**
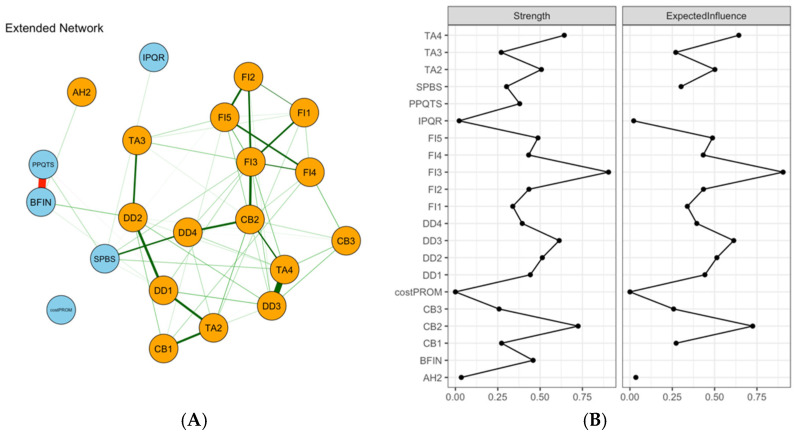
Network analysis visualization for emotional distress with Stress Process Model–related variables (**A**) and centrality metrics (**B**). Note: Green edges indicate positive partial correlations and red edges indicate negative partial correlations; thicker lines indicate stronger absolute edge weights. Centrality plots present z-standardized strength and expected influence (EI). SPBS = Self-Perceived Burden Scale; BFI-N = Neuroticism subscale of the Big Five Inventory; PPQTS = Positive Psychological Capital Questionnaire; IPQR = Illness Perception Questionnaire–Revised; COST-PROM = Comprehensive Score for Financial Toxicity–Patient-Reported Outcome Measure.

In the association network integrating emotional symptoms with Stress Process Model–related variables (Figure 4A), the network included 21 nodes and 69 nonzero edges (out of 210 possible edges). The strongest positive edge in the entire network was observed between self-perceived burden (SPBS) and FI2 (Fatigued) (ω = 0.342). A relatively strong negative edge was observed between CB1 (Confused) and TA3 (Ill at ease) (ω = −0.313). Other notable edges included CB3 (At a loss)-FI4 (Cannot get going) (ω = 0.226), neuroticism (BFI-N)-FI4 (ω = 0.214), and BIPQ-BFI-N (ω = 0.206), alongside connections involving positive psychological capital and depressive symptoms. Centrality analysis suggested that TA2 (Restless) and FI3 (Exhausted) remained relatively central among symptoms, whereas SPBS showed comparatively strong influence among stress-related variables (Figure 4B). Because the CS coefficient for node strength was 0.15 (cor = 0.7), these rankings should be interpreted with substantial caution.

### 3.6. Expanded Network with Quality of Life

**Figure 5:**
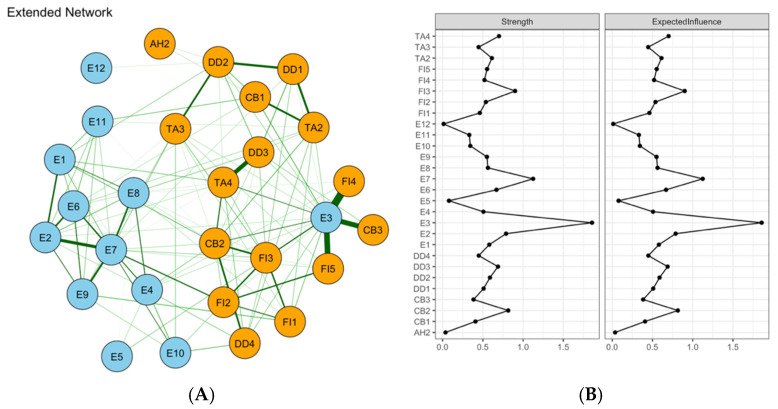
Network analysis visualization for emotional distress with quality of life (**A**) and centrality metrics (**B**). Note: Green edges indicate positive partial correlations and red edges indicate negative partial correlations; thicker lines indicate stronger absolute edge weights. Centrality plots present z-standardized strength and expected influence (EI). E1 = physical functioning; E2 = role functioning; E3 = emotional functioning; E4 = cognitive functioning; E5 = social functioning; E6 = pain; E7 = fatigue; E8 = dyspnea; E9 = appetite loss; E10 = constipation; E11 = nausea; E12 = diarrhea.

A network was constructed to examine conditional associations between emotional distress symptoms and quality-of-life indicators (Figure 5A). This model included 27 nodes and 146 nonzero edges (out of 351 possible edges), indicating extensive interconnections between emotional distress and quality-of-life domains. Functioning domains were mainly linked to fatigue-, depression-, and anxiety-related symptoms, whereas symptom domains also showed close connections with multiple negative emotional symptoms. The strongest edge in the entire network was between FI4 (Cannot get going) and E7 (Fatigue) (ω = 0.485), followed by FI4-E8 (Dyspnea) (ω = 0.405). Within the emotional symptom set, DD1 (Sad) and FI4 showed a prominent connection (ω = 0.367). After covariate adjustment, the residual network still retained several stable links (Figure 5B), suggesting that these associations were not solely driven by demographic or clinical factors. Centrality analysis indicated that emotional functioning (E3) and fatigue (E7) had relatively high centrality within the quality-of-life component, while FI3 (Exhausted) remained among the more central emotional symptom nodes. Because the CS coefficient for node strength was 0.36 (cor = 0.7), these rankings should still be interpreted conservatively.

### 3.7. Network Comparison

Results from the NCT indicated that neither global strength nor overall network structure differed significantly between the high- and low-level groups across SPM-related variables, including illness perception (IPQR), neuroticism (BFI-N), positive psychological capital (PPQTS), financial toxicity (COST-PROM), and self-perceived burden (SPBS) (all *p* > 0.05). These null findings suggest that these variables may be more closely related to overall distress severity than to major changes in network topology, although limited subgroup power and information loss from median splits should be considered. Detailed NCT results are provided in Table 3.

## 4. Discussion

This study provides a theory-informed contextual application of network analysis to emotional distress in gynecologic malignancies. Rather than treating ED as a single total score, we modeled a focused set of frequently endorsed negative mood symptoms and then examined how these symptoms were conditionally linked with demographic/clinical characteristics, SPM-related psychosocial variables, and quality-of-life domains. The manuscript’s primary contribution is therefore contextual and organizational: it shows how an SPM-guided selection of psychosocial correlates can be brought into dialogue with symptom network analysis without claiming that the resulting undirected network is itself a directional stress-process model. Overall, the networks were densely connected and dominated by positive edges, suggesting that emotional symptoms tended to co-occur within a mutually reinforcing system [15,16,17,36]. Within-dimension clustering was evident, but several cross-domain associations also emerged, indicating that fatigue, anxiety, depressive mood, and confusion were not isolated domains.

One of the strongest symptom-level associations was observed between anxiety (TA4) and difficulty initiating activities (FI4). In a cross-sectional network, this edge should be interpreted as a conditional co-occurrence rather than a directional mechanism. Nevertheless, the pattern is clinically plausible: sustained anxious arousal and reduced behavioral activation often coexist during cancer treatment and may be experienced by patients as a linked symptom pair [37,38].

Exhaustion (FI3), restlessness (TA2), and irritability (AH2) showed relatively greater prominence on the centrality indices in the main network. However, because the CS coefficient for the primary network was modest and the expanded networks showed even lower stability, these nodes are better understood as relatively more relationally prominent within this specific network configuration rather than as precise rank-ordered priorities or intervention leverage points. Their prominence should therefore be read as approximate and hypothesis-generating, especially because bootstrapped difference tests among the top nodes were not used to establish statistically reliable rank separation [23]. This caution also reflects current methodological guidance, which suggests that centrality in cross-sectional psychological networks should be interpreted carefully and not translated too directly into clinical action [39,40].

The demographic/clinical expanded network suggested that age group, monthly income, and tumor stage were more connected with emotional symptoms than other background variables, whereas the residual network indicated that the basic symptom-to-symptom structure changed little after covariate adjustment. This pattern suggests that background characteristics may shape overall distress burden without fully determining the internal organization of symptom associations [16].

In the SPM-related network, self-perceived burden showed the strongest direct association with fatigue. This finding is consistent with the idea that distress in cancer is embedded in patients’ role obligations and appraisal processes [19,20]. In the Chinese clinical context, where family-centered caregiving and reciprocity are highly salient, concerns about burdening relatives may be especially emotionally charged. Thus, self-perceived burden may represent an important contextual correlate of fatigue and broader distress, even though the current design cannot establish whether burden drives fatigue, fatigue drives burden, or both.

Illness perception and neuroticism were also conditionally connected to the symptom network, supporting the relevance of cognitive appraisal and vulnerability traits. At the same time, the absence of significant NCT differences between high- and low-risk subgroups deserves substantive emphasis rather than being treated as merely negative. Taken together, these results suggest that psychosocial burden in this sample may influence how much distress is experienced more than it alters how symptoms relate to one another. In other words, the subgroup analyses favor an interpretation of relative structural stability with variation primarily in overall distress severity, rather than clear topological differentiation across psychosocial strata. These null subgroup findings are therefore informative: they indicate either that between-group heterogeneity was smaller than anticipated or that the current sample and median-split approach had limited power to detect subtler topological differences [23]. In this sense, the subgroup analyses function as an empirical boundary test, indicating that psychosocial risk stratification did not translate into clearly distinct symptom architectures in the present dataset.

The network that integrated quality-of-life domains showed extensive links between emotional symptoms and functional outcomes, especially around fatigue-related nodes. The strongest edges connected difficulty initiating activities (FI4) with QLQ-C30 fatigue and dyspnea, supporting the interpretation that fatigue-related impairment may be an important correlational interface between emotional distress and diminished daily functioning [41].

Several methodological issues also temper interpretation. First, the node-screening strategy emphasized clinically frequent negative mood items and included one track based on a core distress indicator. Although screening was restricted to a training set and the final nodes were frozen and validated, the resulting 16-node network is not a neutral slice of the full BPOMS-SF30 but a focused reconstruction of the most prevalent, strongly associated, and selection-stable symptoms. This selection logic likely contributed to the prominence of fatigue- and activation-related items within the retained network. The supplementary sensitivity analysis using the broader 23-item set partly mitigates this concern, but full-scale replication remains important. Second, although the internal consistency of the main scales was acceptable, it was not uniformly strong, which may have attenuated some partial correlation estimates and should be borne in mind when interpreting weaker edges or close centrality rankings. Third, the sample was recruited from a single tertiary hospital and was overwhelmingly Han Chinese, so the findings may not generalize to more diverse populations or different caregiving cultures. Cultural norms around reciprocity, emotional restraint, and family obligation may also shape how self-perceived burden is experienced and reported in this setting. In particular, within many Chinese family systems, concerns about becoming a burden may carry moral and relational meanings that are not fully captured by generic psychosocial constructs; this cultural specificity may partly explain why self-perceived burden was especially salient in the present networks.

Finally, several CS coefficients were low to moderate, especially in the expanded networks. For this reason, we deliberately interpret edge weights and centrality descriptively rather than mechanistically, and we do not claim that relatively central symptoms are causal treatment leverage points [39,40]. In addition, although the archived workflow does not preserve all missing-data details, the analysis assumed that missingness was compatible with a missing-at-random framework for multiple imputation by chained equations. The recoverable aspects of the procedure include the imputation approach itself, the use of predictive mean matching, and the fact that imputation was conducted as a preprocessing step before the exploratory network analyses; however, variable-level missingness summaries, the exact number of imputations, and pooled diagnostic outputs were not retained and should be documented prospectively in future work.

Future studies should replicate these findings in larger multicenter samples, compare the reduced node set with broader symptom networks, and use longitudinal or complementary path-based models when directional hypotheses are of interest. Such work would align with broader methodological agendas calling for replication, clearer theory-model correspondence, and stronger longitudinal designs in network research [42].

### 4.1. Implications for Clinical Practice

From a clinical perspective, the present findings support a systems-oriented monitoring strategy. Symptoms such as exhaustion, restlessness, irritability, and difficulty initiating activities may be especially worth routine descriptive monitoring within this sample because they were relatively more prominent in the estimated networks, not because they can be assumed to be definitive leverage points. Fatigue-related impairment and self-perceived burden also appear to be useful correlates for psychosocial screening. In parallel, the structurally stable subgroup findings suggest that psychosocial stratification may help identify patients with greater distress burden even when the basic architecture of symptom interrelations remains similar. Any clinical prioritization should therefore be considered provisional until replicated in more stable and preferably longitudinal networks.

### 4.2. Limitations

This study has several limitations. First, the cross-sectional, single-center convenience sample precludes causal inference and limits generalizability. In addition, because the sample was predominantly Han Chinese, the findings may not readily generalize to more diverse cultural or clinical settings. Second, several features of the network modeling require cautious interpretation. Some CS coefficients were below recommended levels, especially in the expanded networks, so centrality indices and specific edge estimates should be regarded as approximate rather than precise. In addition, the reduced 16-node network was derived from an exploratory screening pipeline that prioritized frequently endorsed negative symptoms with stronger and more stable associations; consequently, lower-prevalence symptoms from the full BPOMS-SF30 were underrepresented, and the observed network structure should be understood as conditional on this focused selection strategy. Third, some analytic and measurement constraints may also have influenced the findings. The internal consistency of several measures was acceptable rather than strong; subgroup comparisons based on median splits may have reduced statistical power, and although missing data were handled using MICE preprocessing under a working missing-at-random assumption, the archived record did not retain full imputation details, which limits reproducibility.

## 5. Conclusions

In patients with gynecologic malignancies, emotional distress symptoms formed a densely connected, predominantly positive network. Fatigue- and activation-related symptoms were relatively more prominent within the retained symptom system, and self-perceived burden showed a notable conditional association with fatigue. At the same time, the subgroup analyses did not reveal major topological differences across psychosocial strata, suggesting that psychosocial burden in this sample may be reflected more in the severity of distress than in a reorganization of symptom structure. The study’s main contribution is therefore not a causal extension of the SPM, but a theory-informed contextual application of network methods and an empirical boundary test of whether psychosocial stratification corresponds to altered symptom topology.

## Figures and Tables

**Table 1:** General Characteristics of Patients with Gynecologic Malignancies (N = 415).

Variable	Category	n (%)
Age (years)	<50	80 (19.30)
	50–59	127 (30.60)
	60–69	120 (28.90)
	≥70	88 (21.20)
Body Mass Index (kg/m^2^)	<18.5 (Underweight)	20 (4.82)
	18.5–23.9 (Normal weight)	233 (56.14)
	24.0–27.9 (Overweight)	124 (29.88)
	≥28.0 (Obese)	38 (9.16)
Ethnicity	Han	409 (98.55)
	Ethnic minorities	6 (1.45)
Marital status	Unmarried	5 (1.20)
	Married	363 (87.47)
	Divorced/Separated	8 (1.93)
	Widowed	39 (9.40)
Parity	0 births	12 (2.89)
	1–2 births	341 (82.17)
	3–4 births	55 (13.25)
	≥5 births	7 (1.69)
Menopausal status	Natural menopause	344 (82.89)
	Artificial menopause	71 (17.11)
Educational level	Primary school or below	166 (40.00)
	Junior high school	160 (38.55)
	Senior high school/Technical secondary school	48 (11.57)
	College or above	41 (9.88)
Employment status	Employed	105 (25.30)
	Retired	208 (50.12)
	Unemployed	88 (21.20)
	Agriculture	14 (3.37)
Monthly per capita household income (CNY)	<2000	22 (5.30)
	2000–4000	310 (74.70)
	>4000	83 (20.00)
Primary caregiver	Spouse	321 (77.35)
	Children	81 (19.52)
	Other caregivers	13 (3.13)
Type of medical insurance	Employee medical insurance	198 (47.71)
	Resident medical insurance	204 (49.16)
	Self-paid	13 (3.13)
Cancer type	Cervical cancer	182 (43.86)
	Ovarian cancer	123 (29.64)
	Endometrial cancer	83 (20.00)
	Vulvar cancer	9 (2.16)
	Other gynecologic cancers	18 (4.34)
Pathological stage (TNM)	Stage I	137 (33.01)
	Stage II	139 (33.49)
	Stage III	109 (26.27)
	Stage IV	30 (7.22)
Time since diagnosis	0–3 months	80 (19.28)
	3–6 months	124 (29.88)
	6–12 months	80 (19.28)
	1–3 years	64 (15.42)
	≥3 years	67 (16.14)
Comorbidities	Hypertension only	69 (16.63)
	Diabetes mellitus only	43 (10.36)
	Both hypertension and diabetes	52 (12.53)
	Neither	249 (60.00)

**Table 2:** Descriptive consistency of frozen nodes between the training and validation sets.

Node	Item	Training Mean	Training Positive (%)	Validation Mean	Validation Positive (%)
TA3	Ill at ease	2.43	90.3	2.42	88.8
TA2	Restless	2.82	94.8	2.87	96.8
TA4	Anxious	2.41	93.8	2.38	92.8
DD1	Sad	2.37	90.3	2.32	84.8
DD2	Dejected	2.29	90.3	2.27	89.6
DD3	Lonely	2.28	78.3	2.41	83.2
DD4	Blue	2.29	91.7	2.35	91.2
FI1	Worn out	2.93	94.5	2.89	96.8
FI2	Fatigued	3.01	99.0	3.09	98.4
FI3	Exhausted	2.98	95.5	3.04	93.6
FI4	Cannot get going	2.47	82.1	2.37	80.8
FI5	Tired	2.49	94.8	2.52	96.0
CB1	Confused	2.10	76.6	2.19	85.6
CB2	Drowsy	2.39	86.2	2.41	84.0
CB3	At a loss	2.28	86.9	2.20	85.6
AH2	Irritable	1.91	65.9	2.03	74.4

Note: Positive response proportion indicates the percentage of participants with node score ≥ the prespecified threshold.

**Table 3:** Network Comparison Test.

Variable	M	p_M	GS_low	GS_high	S	p_S
SPBS	0.251	0.962	1.0597	0.3069	0.7528	0.6623
BFI-N	0.322	0.430	0.3157	1.5745	1.2588	0.3956
PPQTS	0.179	0.871	0.1054	2.4285	2.3231	0.7622
IPQR	0.340	0.282	0.2812	1.9964	1.7152	–
COST-PROM	0.264	0.935	0.2784	0.5666	0.2881	0.8092

Note: M represents the test statistic for network structure invariance; p_M indicates the corresponding *p* value. GS_low and GS_high represent the global strength of the low- and high-level groups, respectively. S denotes the difference in global strength between the two networks, and p_S indicates the corresponding *p* value. SPBS = Self-Perceived Burden Scale; BFI-N = Neuroticism subscale of the Big Five Inventory; PPQTS = Positive Psychological Capital Questionnaire; IPQR = Illness Perception Questionnaire–Revised; COST-PROM = Comprehensive Score for Financial Toxicity–Patient-Reported Outcome Measure.

**Figure A1:**
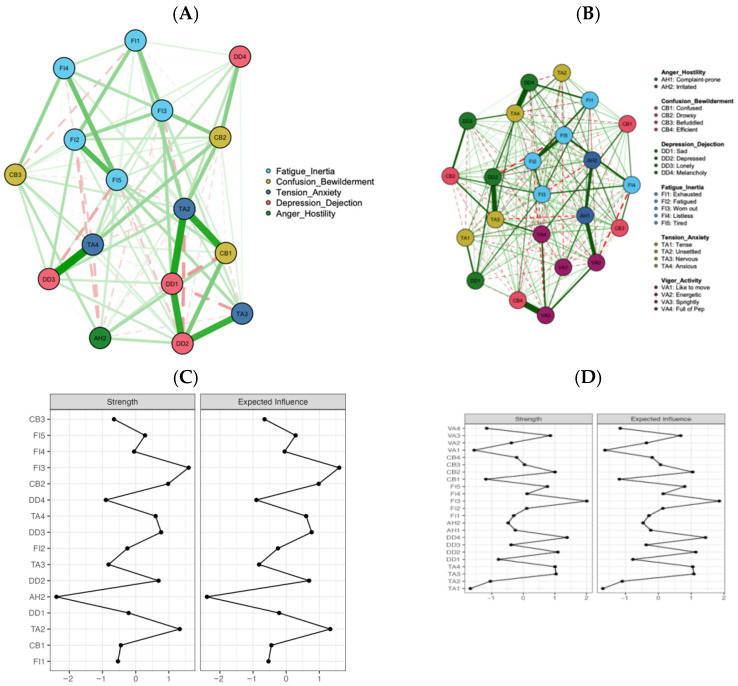
(**A**) Symptom network with 16 screened nodes; (**B**) Symptom network with 23 nodes; (**C**) Centrality analysis for the 16-node net-work; (**D**) Centrality analysis for the 23-node net-work.

## Data Availability

The data supporting the findings of this study are not publicly available due to privacy and ethical restrictions, as they contain sensitive information about study participants. The datasets generated during the current study are available from the corresponding author upon reasonable request.

## Appendix A. Sensitivity Analysis for Node-Selection Robustness

To examine the robustness of the node-screening strategy, we conducted a sensitivity analysis by constructing a 23-node symptom network after excluding only nodes with prevalence < 50%, and compared it with the primary 16-node network reported in the main text. The results were broadly consistent across the two specifications. In the 23-node network, the most central symptoms were still predominantly fatigue-related and anxious-restlessness symptoms, which was largely consistent with the core symptoms identified in the 16-node network. Likewise, the strongest edges in both networks were concentrated in similar symptom pairs, indicating that the principal connection pattern remained stable. Taken together, these findings suggest that although the number of retained nodes varied depending on the screening rule, the core network structure showed good consistency, supporting the robustness of the main conclusions.
